# Natural rice rhizospheric microbes suppress rice blast infections

**DOI:** 10.1186/1471-2229-14-130

**Published:** 2014-05-13

**Authors:** Carla Spence, Emily Alff, Cameron Johnson, Cassandra Ramos, Nicole Donofrio, Venkatesan Sundaresan, Harsh Bais

**Affiliations:** 1 Department of Biological Sciences, University of Delaware, Newark, USA; 2 Delaware Biotechnology Institute, Newark, USA; 3 Department of Plant and Soil Sciences, University of Delaware, Newark, USA; 4 Department of Plant Biology, University of California, Davis, USA

**Keywords:** Rice, Blast, *Magnaporthe oryzae*, *Psuedomonas*, Hydrogen cyanide (HCN), Biocontrol, Induced systemic resistance

## Abstract

**Background:**

The natural interactions between plant roots and their rhizospheric microbiome are vital to plant fitness, modulating both growth promotion and disease suppression. In rice (*Oryza sativa*), a globally important food crop, as much as 30% of yields are lost due to blast disease caused by fungal pathogen *Magnaporthe oryzae*. Capitalizing on the abilities of naturally occurring rice soil bacteria to reduce *M. oryzae* infections could provide a sustainable solution to reduce the amount of crops lost to blast disease.

**Results:**

Naturally occurring root-associated rhizospheric bacteria were isolated from California field grown rice plants (M-104), eleven of which were taxonomically identified by16S rRNA gene sequencing and fatty acid methyl ester (FAME) analysis. Bacterial isolates were tested for biocontrol activity against the devastating foliar rice fungal pathogen, *M. oryzae* pathovar 70–15. *In vitro*, a *Pseudomonas* isolate, EA105, displayed antibiosis through reducing appressoria formation by nearly 90% as well as directly inhibiting fungal growth by 76%. Although hydrogen cyanide (HCN) is a volatile commonly produced by biocontrol pseudomonads, the activity of EA105 seems to be independent of its HCN production. During *in planta* experiments, EA105 reduced the number of blast lesions formed by 33% and *Pantoea agglomerans* isolate, EA106 by 46%. Our data also show both EA105 and EA106 trigger jasmonic acid (JA) and ethylene (ET) dependent induced systemic resistance (ISR) response in rice.

**Conclusions:**

Out of 11 bacteria isolated from rice soil, pseudomonad EA105 most effectively inhibited the growth and appressoria formation of *M. oryzae* through a mechanism that is independent of cyanide production. In addition to direct antagonism, EA105 also appears to trigger ISR in rice plants through a mechanism that is dependent on JA and ET signaling, ultimately resulting in fewer blast lesions. The application of native bacteria as biocontrol agents in combination with current disease protection strategies could aid in global food security.

## Background

With a burgeoning world population, food security and crop protection are of utmost importance. One of the most important staple food crops is rice, which over 3.5 billion people are dependent on for daily energy consumption. Rice blast disease, caused by the wide-spread foliar fungal pathogen *Magnaporthe oryzae*, occurs in more than 85 countries and causes devastating crop loss. Each year this disease destroys enough rice to feed an estimated 60 million people [1] and, unfortunately, there are currently no effective means to provide lasting, adequate control of the pathogen.

Current low cost protection strategies include planting of uninfected seeds, limiting nitrogen fertilizers, perpetual field flooding, and post-harvest burning of plant remains [2]; however, these strategies can neither eliminate infections nor resolve situations when a field does become infected. Rice varieties with genetic resistance to rice blast, for example, a cultivar carrying the *Pi-ta* R-gene are effective in initiating a gene-for-gene interaction with the corresponding *M. oryzae* avirulence (AVR) gene and conferring resistance; yet the pathogen rapidly overcomes plant-encoded resistance [3,4]. Chemical pesticides offer marginal protection from the disease, yet pose environmental risks and may put non-pathogenic organisms, including humans, at risk [5]. Thus, the control strategies currently employed are limited in effectiveness and may lead to further problems. An alternative means of crop protection would be through the use of biological control agents (BCA).

An effort is underway to describe the microbiome that associates with plants and their impact on plant health and productivity. As with the gut microflora in humans, rhizospheric microbial communities aid in nutrient acquisition and control soil pathogens through competition for nutrients and production of antimicrobials [6]. Some gram-negative *Pseudomonas* species are well-studied biocontrol bacteria that have been shown to produce a number of antimicrobial secondary metabolites [7]. These include but are not limited to phenazines [8], hydrogen cyanide [9,10], 2,4-diacetylphloroglucinol [11], pyrrolnitrin [12], and pyoluteorin [13], as well as the cyclic lipopeptides tensin [14] and viscosinamide [15]. The most well studied Gram-positive biocontrol bacteria are within the genus *Bacillus*, and have been shown to produce low molecular weight surfactins with antifungal activity [16] as well as antifungal lipopeptides called kurstakins [17].

BCA also help protect plants against foliar pathogens by altering of host immunity for quicker defense responses. This induced systemic resistance (ISR) response occurs through root to shoot long distance intra-plant signaling, priming the plants to better resist pathogen attack [18]. In most cases ISR depends on jasmonic acid (JA) and ethylene (ET) plant signaling and not salicylic acid (SA) signaling as seen with systemic acquired resistance [19]. Priming occurs when the plant recognizes microbial cell components, secretions, or volatiles [20]. Upon attack by a pathogen, primed plants have more rapid cellular defense responses [21]. This is due to increased accumulation of inactive transcription factors as a response to microbial colonization, that are then activated during pathogen attack, creating enhanced expression of defense genes [22]. *Pseudomonas fluorescens* strain WCS417r was the first bacterium documented to induce a systemic response in carnation (*Dianthus caryophyllus* L.) allowing it to be more resistant to *Fusarium* wilt [23].

Schroth et al. [24] described how plants grown in certain soils are less prone to disease. These disease-suppressive soils can occur naturally due to their physiochemical properties promoting colonization of biological control (hereafter biocontrol) microbes, or can be established through plant recruitment of beneficial microbes to the roots, regardless of soil type, when under biotic stress. For example, *Arabidopsis thaliana* infection by the foliar bacterial pathogen *Pseudomonas syringae* pv *tomato* DC3000 (hereafter DC3000) induces root secretion of _L_-malic acid, which attracts the beneficial rhizobacterium *Bacillus subtilis* FB17 to the roots [25,26]. FB17 then triggers the expression of defense-related genes in *A. thaliana* leaves, including pathogenesis-related protein PR1 and plant defensin PDF1.2, reducing DC3000 growth and disease incidence [25,26].

Understanding and manipulating natural associations between rice plants and their rhizospheric communities, in combination with current disease control strategies, would be a comprehensive and effective way to reduce infection and increase food production. The objective of this study is to isolate and characterize naturally occurring and closely associated rhizospheric rice bacteria in order to identify possible biocontrol bacteria for *M. oryzae*. The bacteria and bacteria-derived components could then be used as fungal suppressors. We have identified a *Pseudomonas* isolate, EA105, which appears to inhibit *M. oryzae* through direct antagonism as well as through the induction of systemic resistance in rice.

## Results

### Isolation and identification of rhizobacteria

**Figure 1:**
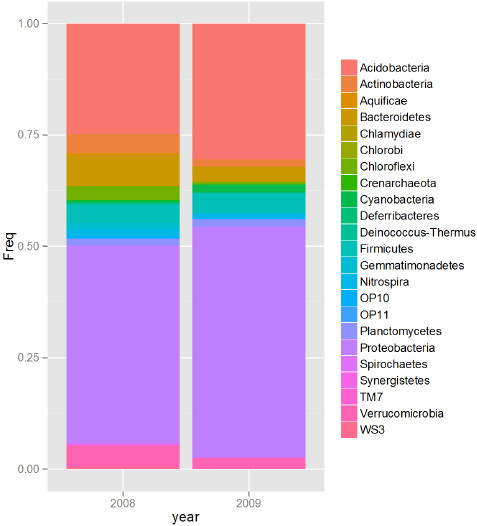
**Relative abundance (frequency) of the major bacterial phyla present in the rice rhizosphere microbial community recorder over two-years.** The frequencies shown were obtained via classification of 16S rDNA sequences corresponding to a total of 654 and 630 clones, for 2008 and 2009 respectively.

Rhizospheric soil samples from California field-grown M-104 rice plants were sequenced for bacterial 16S rDNA and distributions of the phyla (Figure 1) and genera (Additional file 1: Figure S1) of bacteria present in the soil samples were determined. There were 8 to 10 phyla (among Acidobacteria, Actinobacteria, Bacteroidetes, Cyanobacteria, Firmicutes, Gemmatimonadetes, Nitrospira, Planctomycetes, Proteobacteria, Verrucomicrobia) that were considered abundant for the 2008 and 2009 data respectively (Figure 1). For these, the 16S rRNA sequences each individually make up greater than 1% of the total. Apart from the Proteobacteria that make up 44% and 50% of the 16S sequences, the second-most abundant phylum was Acidobacteria making up 24% and 30% of the sequences in the 2008 and 2009 samples respectively. Other phyla making up greater than 4% of the sequences were Actinobacteria, Bacteroidetes and Firmicutes. At the rank of genera, the top 1% of sequences (99th percentile) were comprised of Acidobacteria subdivisions Gp1, Gp3, Gp4, and Gp6, and also Nitrosospira, a member of the Betaproteobacteria (Additional file 1: Figure S1). From the same soil samples, naturally occurring root-associated and root-bound rhizospheric bacteria were isolated (Table 1). Strains labeled EA101-EA108 were isolated on TY agar, and strains labeled EA201-EA202 were isolated on LB agar. One bacterium, labeled EA303, was isolated using *Chlorobium* plating (CP) agar plates with benzoate as the sole carbon source. A total of eleven isolates were taxonomically identified by fatty acid methyl ester (FAME) analysis and their identities were further confirmed using 16S rRNA gene sequencing (Table 1). Six out of the 11 isolates belonged to the class *Gamma-proteobacteria*, and of these, 5 were of the genus *Pseudomonas*. This may be due to their ability to be cultured and their natural abundance in the soil environment, including the rhizosphere.

**Table 1:** **Identification of rice soil isolates. List of rhizospheric bacteria isolated from rhizosphere of *O. sativa *** **cultivar M-104 and identified by 16S rRNA gene sequencing and fatty acid methyl ester (FAME) analysis**

**Genus**	**Species**^**a**^	**Similarity Index**	**Confidence Level**	**Strain Label**	
*Pseudomonas*	*Corrugata*	0.761	Species inconclusive	EA104	Root associated
	*Chlororaphis*	0.598	Genus	EA105	Root
	*Chlororaphis*	0.77	Species	EA107	Root
	*Putida*	0.785	Species	EA108	Root
	-	0.232	No match*	EA303	Root associated
*Pantoea*	*Agglomerans*	0.896	Species	EA106	Root
*Dyadobacter*	-		Genus*	EA202	Root associated
*Pedobacter*	*Heparinus*	0.682	Species	EA101	Root associated
*Chryseobacterium*	*Balustinum*	0.776	Species	EA102	Root associated
*Rhodococcus*	*Rubripertincta*	0.807	Species	EA103	Root associated
*Arthrobacter*	*Oxydans*	0.758	Species	EA201	Root associated

^a^Closest match in MIDI library as determined by FAME analysis.

- Inconclusive match.

*Genus solely determined by 16S rRNA gene sequencing.

### In vitro antifungal properties of rice rhizospheric bacterial isolates

**Figure 2:**
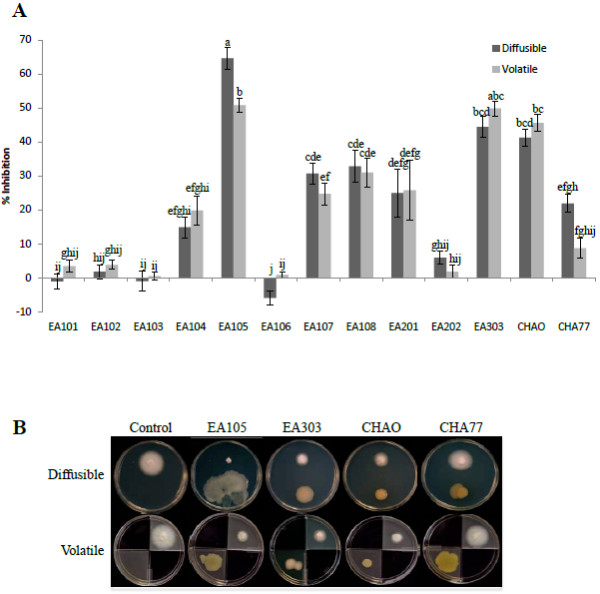
**Inhibition of *M. oryzae *vegetative growth by rice soil isolates. A)** Antimicrobial assay showing the degree of inhibition of *M. oryzae* 70–15 by naturally isolated rice rhizobacteria as well as *P. fluorescens* CHAO and cyanide mutant CHA77. Error bars indicate standard error. Different letters indicate statistically significant differences between treatments (Tukey’s HSD). **B)** Representative images of the fungal inhibitory effect seen when 70*–*15 was exposed to bacterial diffusible and volatile compounds (diffusible plates), or solely through volatile compounds (volatile plates).

The effect of naturally associated rice rhizobacteria (see Table 1) on growth and development of *M. oryzae* strain 70–15 was assessed using petri dish assays. A diffusible assay evaluated the effect, if any, of bacterial-derived diffusible compounds on *M. oryzae* 70–15 (hereafter 70–15) without direct contact. The two microbes could communicate and interact through both volatile compounds and diffusible compounds. All isolates were tested and five *Pseudomonas* isolates (EA104, EA105, EA107, EA108, and EA303) showed significant inhibition of 70–15 growth (Figure 2A). The most dramatic effect was seen by the *Pseudomonas* isolate EA105, inhibiting fungal growth by 65% after 5 days, relative to the control (Figure 2A).

Bacterial volatiles have been receiving increasing attention for their roles not only as odors, but as phytostimulators, antimicrobials, and compounds involved in inducing a systemic resistance response as well [27-29]. To examine whether volatile antifungal metabolites were playing a role in the observed hindering of 70–15 growth, a volatile (compartment) plate assay was performed using petri dishes that were divided into four quadrants. *M. oryzae* and rice bacterial isolates were placed in opposite compartments where they shared the same headspace, yet there was no exchange of diffusible compounds. Any inhibition observed was therefore due to volatile compounds. All of the *Pseudomonas* isolates significantly reduced growth to about the same degree as seen in direct plates, except for EA105, whose inhibition effect was reduced in compartment plates (Figure 2A). Bacterial motility allows for a number of beneficial activities, including acquiring more nutrients, maneuvering away from toxic substances, and colonizing in optimal environments [29]. EA105 is able to spread across plates quickly through swimming and swarming (Additional file 2: Figure S2) and restriction to one quadrant of a plate could have contributed to the reduction in inhibition. A similar reduction in EA105’s inhibitory activity was seen when EA105 was grown on CM agar instead of LB agar, and in liquid culture as opposed to agar (Additional file 3: Table S1).

To see if metabolically active cells are needed for the direct antagonism exhibited by EA105, a control experiment was performed using the same diffusible assay set-up, except heat killed EA105 cells or the spent media (cell-free supernatant) were used in place of live cells. Neither the heat killed cells nor the spent media showed any significant effect on fungal growth (Additional file 4: Figure S3A), indicating that active cells are needed for fungal inhibition. To further examine the nature of EA105-derived inhibition, *M. oryzae* 70–15 plugs were taken from plates where 70–15 had been exposed to EA105 (inhibited) and were subcultured onto fresh CM agar. When no longer exposed to the bacteria, 70–15 grew normally (Additional file 4: Figure S3B), indicating the fungistatic nature of EA105.

One frequently reported toxin produced by some pseudomonad species is hydrogen cyanide (HCN), which binds to cytrochome c oxidase and blocks cellular respiration [30]. HCN can exist in both a gaseous or aqueous state, suggesting that it can be released by the bacteria as a volatile, as well as secreted into the media. Therefore, we tested the tolerance of 70–15 to a known cyanide (CN) producer, *Pseudomonas fluorescens* CHAO [31], and its HCN production negative mutant, *P. fluorescens* CHA77 [32]. In diffusible plates, CHAO significantly reduced fungal growth by 46% (Figure 2A); however, this was not as drastic of an inhibition effect as seen by EA105. CHA77 also significantly reduced fungal growth, but only by 22% directly and 10% through volatiles (Figure 2A).

**Figure 3:**
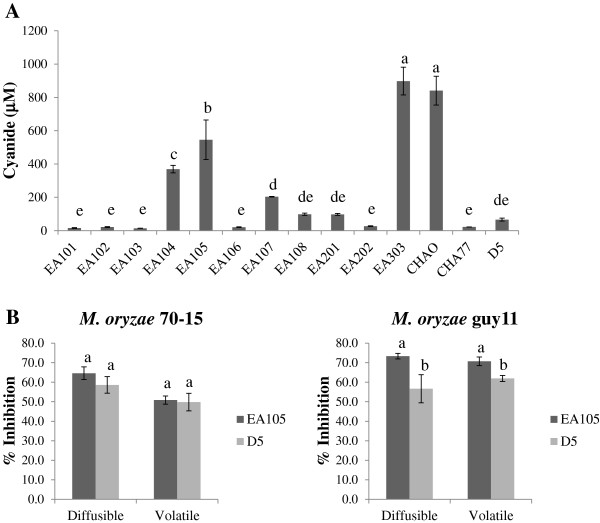
**Cyanide production by rice isolates and activity of cyanide mutant D5 against *M. oryzae. *A)** Bacterial cyanide production of all rice isolates, D5, CHAO, and CHA77 was measured after 24 hour incubation using the Lazar Model LIS-146CNCM micro cyanide ion electrode. Different letters indicate statistical significance (Tukey’s HSD). **B)** Antimicrobial assay against *M. oryzae* strain 70–15 and its parental strain guy11 with EA105 and its cyanide deficient mutant, D5. Different letters indicate statistical significance (Tukey’s HSD).

Since many of the known pseudomonads, including *P. fluorescens* strain CHAO [31], produce CN as a major antimicrobial component, bacterial CN production in stationary phase culture supernatants of all rice isolates was quantified using the Lazar Model LIS-146CN-CM micro cyanide ion electrode [33]. As controls, we also measured CN generated by *P. fluorescens* CHAO and CHA77. EA105 produced around 500 μM cyanide after 24 hours of incubation, while EA303 and CHAO produced around 700 μM (Figure 3A). As expected, CN production was severely diminished in CHA77, which has a disrupted CN biosynthesis operon (Figure 3A). Even though EA105 produces less cyanide, it inhibits *M. oryzae* vegetative growth more than CHAO, indicating the involvement of other antifungal metabolites.

Additionally, a HCN biosynthetic mutant, D5, was created in EA105 in which the *hcn*ABC operon involved in CN synthesis was disrupted and CN generation was diminished (Figure 3A). The two plate-based bioassays were utilized to evaluate the importance of CN in EA105 antibiosis against 70–15. Our data show that EA105 and the D5 mutant attenuate the growth of 70–15 and guy11 to a similar degree under both diffusible and volatile assays (Figure 3B). CHAO’s cyanide deficient mutant, CHA77, shows a drastic reduction in ability to inhibit *M. oryzae* (Figure 2A), while EA105’s cyanide deficient mutant, D5, only shows minimal reduction in antifungal activity, suggesting that EA105 and CHAO have different mechanisms of antibiosis. This also indicates that the restriction of *M. oryzae* growth by EA105 is mainly independent of CN, and requires an unidentified bacteria-derived compound.

Both organic and inorganic volatile compounds produced by bacteria have been shown to provide biocontrol activity against plant pathogens [34,35]. To determine whether the antifungal activity seen by EA105 volatiles are due to organic or inorganic compounds, or both, the volatile (compartment) plate design was used. As previously described, *M. oryzae* 70*–*15 and the bacteria were placed in opposite compartments; however, the two remaining compartments were filled with activated charcoal/carbon, which will adsorb organic bacterial volatiles. The plates amended with activated charcoal showed normal fungal growth and no inhibition through bacterial volatile compounds (Additional file 5: Figure S4). This implies that the active antifungal volatiles are organic compound(s), and henceforth referred to as volatile organic compounds (VOCs).

**Figure 4:**
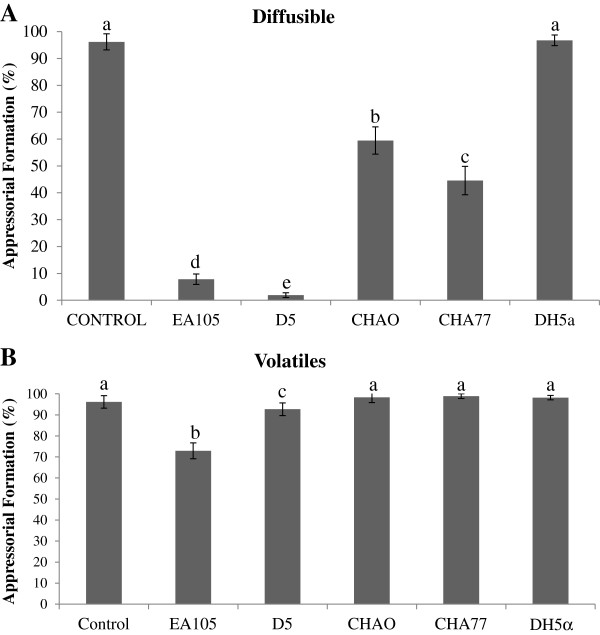
**Inhibition of M. oryzae appressoria after bacterial treatment.** Effect of bacteria on *M. oryzae* 70–15 appressorial formation through **A)** direct bacterial treatment, or through **B)** indirect (or volatile) bacterial treatment. Germinated conidia were incubated in a 50uL drop with bacterial treatment (EA105, cyanide mutant D5, CHAO, cyanide mutant CHA77, or E. coli DH5α) or placed in a drop next to the bacterial treatment for the indirect assay. Error bars represent standard deviation. Different letters indicate a significant difference (Tukey’s HSD).

In addition to the effect rhizobacterial isolates have on vegetative growth, these bacteria also affect development of conidia into a specialized infection structure called the appressorium. During pathogenesis, a penetration peg develops at the tip of the appressoria, which enables physical puncturing of the plant cuticle and infection of the host [36]. EA105 inhibited 70–15 appressorial formation by nearly 90% compared to the control; while a known biocontrol strain of *P. fluorescens*, CHAO, inhibited about 60% through direct treatment (Figure 4A). An unexpected observation was that both cyanide mutants, D5 and CHA77, inhibited appressorial formation slightly more than their cyanide-producing counterparts, EA105 and CHAO, respectively. Although it has not been shown in fungi, there is evidence that sub-lethal concentrations of cyanide can trigger defense mechanisms in nematodes [37]. Through indirect treatment, CHAO completely failed to inhibit appressorial formation while EA105 was still able to reduce appressorial formation by about 20% (Figure 4B). This indicates that volatile compounds may be involved in the inhibition of vegetative growth as well as in the reduction of appressorial formation in the case of EA105.

**Figure 5:**
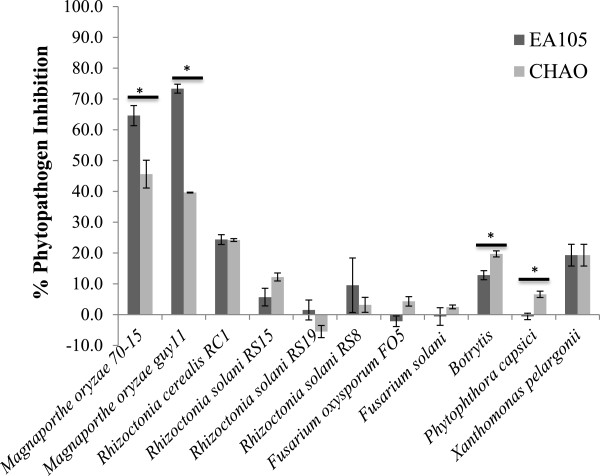
**Activity of EA105 against naturally isolated phytopathogens.** Inhibition of naturally isolated phytopathogens by EA105 and CHAO in comparison to *M. oryzae.* With the exception of lab strain *F. oxysporum* FO5, all pathogens were isolated from infected plants or soil, and acquired from Nancy Gregory at the University of Delaware. Error bars represent standard error. Asterisks indicate significant differences between EA105 and CHAO treatment (Student’s t-test, p < 0.05).

To gain a better understanding of the effectiveness of EA105’s antimicrobial potential against diverse phytopathogens, EA105 was tested against a variety of naturally isolated pathogens. Both EA105 and CHAO inhibited other phytopathogens to a similar and lesser degree than *M. oryzae*; however EA105 was able to restrict *M. oryzae* growth to a significantly greater degree than CHAO (Figure 5). This suggests the antimicrobial activity seen by EA105 is more specific and effective against a rice pathogen compared to other non-specific pathogens.

### Characterization of antifungal metabolites from EA105

Volatile organic compounds (VOCs) produced by EA105 were identified using solid-phase microextraction-gas chromatography mass-spectrometry (SPME-GC-MS) (Table 2). The most abundant peak in the headspace profile of EA105 was identified as 1-undecene, being produced at a concentration of 270 μM over 24 hours based on commercial standards (Additional file 6: Table S2; Additional file 7: Figure S5A). Past antimicrobial studies with 1-undecene shows it has no effect on *Sclerotinia sclerotiorum*[34] and a small effect on *Fusarium culmorum*[38]. S-methyl thioesters were also identified in the volatile profile of EA105, producing around 30 μM in 24 hours (Additional file 6: Table S2; Additional file 7: Figure S5A). Antifungal activity against 70–15 by these compounds was examined and no significant growth reduction was seen at biologically relevant concentrations (Additional file 7: Figure S5B-C), suggesting these compounds are not the bioactive volatiles produced by EA105 as an antifungal.

**Table 2:** **List of volatile organic compounds (VOCs) identified in *Pseudomonas *** **isolate EA105 headspace by GC-MS**

**RT (min)**	**Compound**
***Alcohols***	
14.07	2-Undecanol
***Hydrocarbons***	
7.28	Cyclopropane, 1-methyl-2-pentyl-
10.77	1,4-Octadiene
10.89	1-Undecene
12.42	1-Dodecene
13.71	Cyclodecene
13.91	1-Tridecene
***Ketones***	
13.94	2-Undecanone
16.67	2-Tridecanone
***S-containing compounds***	
3.72	Methyl thiolacetate
4.5	Dimethyl disulfide
5.54	S-methyl propanethioate
8.27	S-methyl 3-methylbutanethioate

Although not directly correlated to vegetative growth reduction, we were interested to see if EA105-derived thiol-esters could reduce virulence; therefore the effect on 70–15 conidial germination and ability to form appressorium was examined post EA105 treatment. Even though a large effect was not seen, there was significant reduction in appressorial formation by all compounds at 100 μM concentration (Additional file 8: Table S3).

### EA105 treatment to rice roots primes resistance against M. oryzae

**Figure 6:**
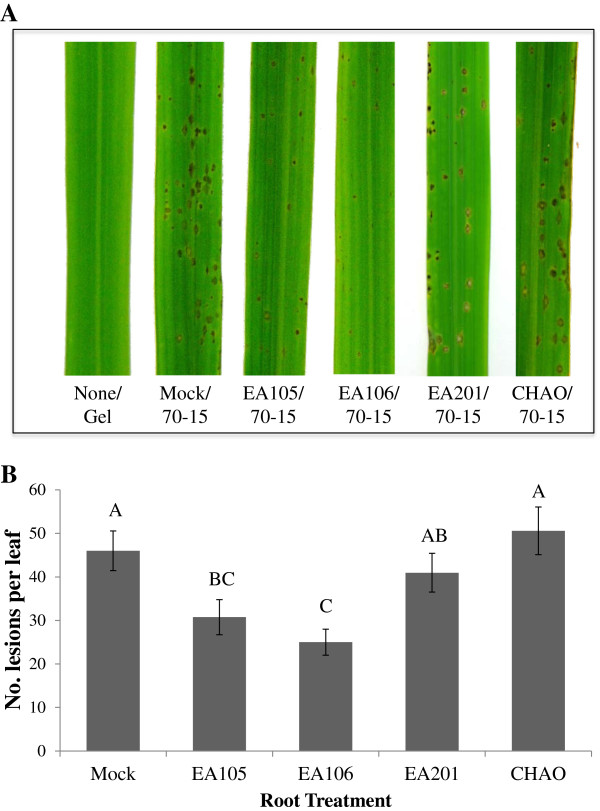
**The effect of rhizobacterial priming on rice blast lesion formation.** Spores were sprayed on 3-week old whole plants 24 hour after being root primed with mock, EA105, EA106, EA201 or CHAO suspension. **A)** Representative leaf segments of mock or rhizobacterial primed plants. **B)** The average number of lesions formed on the second youngest leaf of *O. sativa* cv. Maratelli. Error bars indicate standard error. Means with the same letter do not differ significantly (Tukey’s HSD).

Induced systemic resistance (ISR) is elicited by plant growth promoting rhizobacteria (PGPR) and results in increased disease resistance in plants. Our data previously showed that EA105 directly inhibits fungal growth by the production of an antifungal compound. Next, we tested if EA105 could also suppress *M. oryzae* indirectly by inducing changes in the host plant. Three-week old roots of soil-grown rice cv. Maratelli (highly susceptible to *M. oryzae*) were root inoculated with rhizobacteria and after 24 hours, the plants were challenged with *M. oryzae* 70–15 spores. In addition to EA105, rice isolates EA106, a *Pantoea agglomerans*, and EA201, an *Arthrobacter oxydans*, were also tested (see Table 1). Strikingly, the plants whose roots had been pretreated, or 'primed’, with EA105 and EA106 showed a significantly reduced number of blast lesions (*P* ≤ 0.0087 and 0.0003, respectively), as compared to the plants receiving no pretreatment (Figure 6). Interestingly, pretreatment with a previously characterized direct antagonist of *M. oryzae*, *P. fluorescence* CHAO [39], conferred no protection against disease formation on the leaves (Figure 6). Although it has previously been reported that CHAO induces ISR in *Arabidopsis thaliana*[40], rice is a non-native host of CHAO, being originally isolated from Swiss soils suppressive to black root rot [41]. These results clearly support the hypothesis that root colonization by EA105 and EA106 induces plant-encoded mechanisms which prime rice for foliar attack by *M. oryzae*, enhancing a defense response which leads to reduction of *M. oryzae* infection on the aerial portion of the plant.

**Figure 7:**
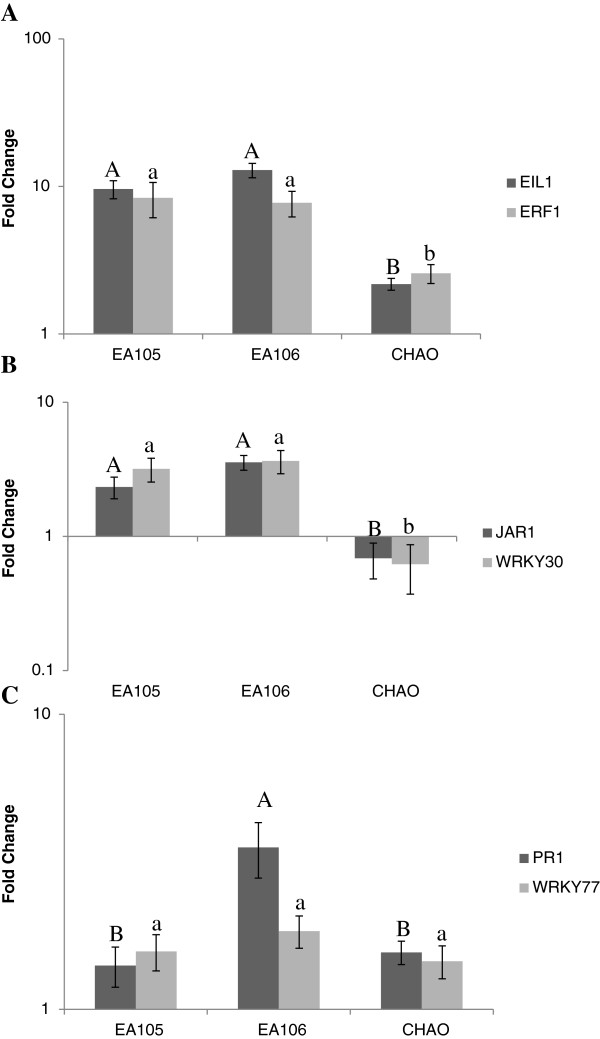
**Expression of defense related genes in rice plants treated with rhizobacteria EA105 and EA106.** Roots of aseptically grown rice plants were treated with EA105 or EA106. Leaf samples were collected at 24 hrs post treatment and the expression of genes involved in **A)** ethylene, **B)** jasmonic acid (JA), or **C)** salicylic acid (SA) signaling was examined. Error bars indicate standard error. Means with the same letter do not differ significantly (Tukey’s HSD).

To further explore the mechanism by which isolates EA105 and EA106 reduce lesions through a plant-mediated mechanism, the expression of several key ISR genes were examined in rice at 24 hours post bacterial treatment. As a control, we also examined the effect of CHAO, which does not reduce the number *M. oryzae* lesions on rice plants. With EA105 or EA106 treatment, there was significant up-regulation of the JA responsive genes, *JAR1* and *WRKY30,* while CHAO treatment down regulated these genes. Similarly, ET responsive genes, *EIL1* and *ERF1,* were also up-regulated with EA105 and EA106 treatment, but to a significantly lesser extent with CHAO treatment (Figure 7). A positive control with JA (50 μM) treatment also induced JAR1 and WRKY30 (data not shown). There was only slight induction of SA responsive genes PR1 and WRKY77 with the bacterial treatments (Figure 7C). The SA responsive genes were also induced by SA treatment (1 mM) (data not shown). Of the 6 genes examined, expression patterns were similar between EA105 and EA106 treatments for all genes except PR1. In rice treated with EA106, there was a significantly stronger induction of PR1 than in rice plants treated with EA105. The data suggest that EA105 induces a JA and ET dependent ISR that may protect plants against *M. oryzae*.

## Discussion

In order to make a significant impact on global food security, a biocontrol solution to rice blast disease must be developed that is both effective and sustainable while reducing or eliminating the need for synthetic chemical fungicides. We have found microbes from the rice rhizosphere that attenuate *M. oryzae in vitro* and *in planta*. Most notable is *P. chlororaphis* strain EA105, which has demonstrated the ability to severely restrict the growth of rice pathogen *M. oryzae,* and is therefore a strong candidate for a novel biocontrol agent against rice blast disease. Previously, *P. chlororaphis* isolates have been shown to be agriculturally important in the biocontrol of several plant pathogens including *Sclerotinia sclerotiorum*[42], *Rhizoctonia cerealis*[43], *Seiridium cardinale*[44], and *Leptosphaeria maculans*[45]. To our knowledge, this is the first report of *P. chlororaphis* reducing rice blast symptoms. In contrast to chemical fungicides, biocontrol bacteria produce a mixture of antifungal compounds which can fluctuate based on environmental cues [46]. The fungistatic activity of EA105 could lead to a longer-term, more effective strategy for reducing rice blast disease than current chemical fungicides, which exert stronger selective pressure for *M. oryzae* to develop resistance. Furthermore, as living organisms, these biocontrol microbes are continuing to evolve with their rhizospheric neighbors ensuring a more sustainable solution.

To gain a better understanding of the composition and diversity of the rice rhizospheric soil, we used a metagenomic approach to examine the phyla and genera that naturally inhabit this niche. Distribution of phyla was consistent across growing seasons, with the two predominant phyla being Acidobacteria and Proteobacteria. Acidobacteria have only recently been discovered and the vast majority are currently unculturable. However, their abundance in soil has been documented, and they may be playing a crucial role in the rhizosphere that has yet to be determined [47]. Proteobacteria is a very broad phylum, encompassing a variety of bacteria, including Pseudomonads which are gamma-proteobacteria [48].

Evidence shows that stress to the aerial portions of plants can stimulate rhizo-deposition of chemo-attractants to enhance colonization by rhizobacteria [26,27]. Effective plant defense may be due to an ability of the host plant to modulate the composition of root exudates, attracting microbes which can trigger plant resistance. The recruitment of beneficial microbes can also alter physiological functions in plants to resist aerial pathogens [49]. Although *M. oryzae* is most commonly a foliar pathogen, it also has the ability to infect roots [50,51] and is closely related to other root pathogens such as *M. poae, M. rhizophila*, and *Gaeumannomyces graminis*[51]. Root infection by *M. oryzae* is often followed by dispersal to the shoots and traditional blast lesion formation [51]. Therefore, the direct antifungal activity of EA105 against *M. oryzae* could have ecologically relevant implications in preventing blast infections.

Our data reveal that treatment of soil-grown rice plants with EA105 activates basal resistance mechanisms against 70–15 *in planta*. The precise mechanism by which rice rhizospheric microbes induce physiological effects on the host (rice) is not known, although some of these changes are modulated through the signaling of small molecules such as salicylic acid (SA), jasmonic acid (JA), or ethylene (ETH) [52]. The pathogenesis related, or PR, genes such as PR1 and WRKY77 are SA responsive [53] and are up-regulated during pathogen infection, ultimately triggering a defense response and reducing disease symptoms [54]. However, beneficial rhizobacteria such as *P. fluorescens* WCS374r have been shown to stimulate a defense response which induces resistance in rice to *M. oryzae*, but is completely independent of SA signaling [55]. Similar to this finding, our gene expression data suggest that EA105 triggers ISR in rice through a mechanism that involves both JA and ETH and to a lesser extent SA signaling. The JA responsive genes JAR1 and WRKY30 are crucial to JA signaling and are required for the stimulation of ISR in *A. thaliana* as well as rice [56,57] and both of these genes were highly expressed 24 hours after EA105 and EA106 treatment but not with CHAO treatment. We saw similar up-regulation of the ethylene responsive genes EIL1 and ERF1, which have also been implicated in ISR signaling and reduction in disease susceptibility [58]. Moreover, we demonstrate the ability of EA105 to severely restrict mycelial growth of 70–15 and almost completely halt appressorium formation on abiotic hydrophobic surfaces. This suggests that the beneficial microbiome of rice could attenuate the virulence of rice blast through multiple mechanisms; therefore, manipulation of the rhizosphere is a valuable and comprehensive manner in which to target biotic stresses.

Biocontrol agents are currently employed to control rice pathogens that cause fungal sheath blight [59-63] and a subset of fungal pathogens that cause rice blast [64,65]. With a few exceptions [66,67] the biocontrol agents tested were not isolated from rice, as compared to the bacterial strain EA105, which was isolated from the rice rhizosphere. We speculate that a microbe which is confirmed to associate with field grown rice roots, such as EA105, may have better implications for rice protection compared to unrelated biocontrol isolates due to its ability to compete and survive in the rice rhizosphere. Previous studies have shown a relative of Psuedomonas, *Delftia tsuruhatensis*, to directly inhibit *M. oryzae* and also reduce lesions in rice by about 50%, however the mechanism of lesion reduction has not been examined [66]. Isolates from the rice and millet rhizospheres, including 13 Bacilli and 6 Psuedomonads, did show direct inhibition and lesion reduction of Setaria blast, on the host plant Foxtail millet (*Setaria italica* L) though these isolates were not tested in rice [68]. There have also been reports of naturally isolated rice rhizobacteria reducing blast in aerobically grown rice in Brazil, though the isolates have not been identified and the mechanism by which they induce resistance has not yet been examined [69]. Similarly, Naureen et al. investigated multiple isolates from bulk soil and the rice rhizosphere for their direct antagonism against *M. oryzae* and their ability to reduce lesions *in planta*, but the mechanisms underlying these activities have not yet been explored. Five of the isolates examined were *Pseudomonas sp.* but these 5 isolates were from bulk soil rather than the rice rhizosphere [67]. Two isolates from the rhizosphere of *Lupinus hispanicus*, *Pseudomonas fluorescens* Aur 6 and *Chryseobacterium balustinum* Aur 9, showed the ability to reduce blast severity and increase rice production when co-inoculated [70] however, these isolates were not originally isolated from the rice rhizosphere and the way in which they reduce lesions has not yet been described. De Vleesschauwer et al. [55], thoroughly examined the way in which *P. fluroescens* WCS374r induces resistance in rice, independent of SA signaling, and mediated through the ETH and octadecanoid pathways. Strain WCS374r is a spontaneous rifampycin mutant of lab strain WCS374 [55]. De Vleesschauwer et al. provide valuable insight into the mechanisms underlying ISR against *M. oryzae*, and we have shown that a natural rice isolate, EA105, shows parallels in its ability to trigger ETH signaling while minimally impacting SA signaling. We have, in a way, combined these stories to investigate how a natural rice isolate works in reducing blast both through direct and plant-mediated mechanisms.

Shimoi et al. [71], examined a novel mechanism of blast reduction by selectively isolating phyllospheric microbes from rice, including one *P. geniculata* strain, which catabolizes collagen and gelatin. Some of these microbes were able to reduce blast symptoms when co-inoculated onto rice leaves, presumably by disrupting the adhesion of the spore tip mucilage and extracellular matrix from the leaf surface, preventing proper attachment by *M. oryzae*[71]. It would be interesting to test such a method in combination with a root-associated microbe such as EA105, which can induce resistance through plant based signaling.

Thorough groundwork has been laid in testing methods for introducing biocontrol bacteria to plants. Talc-based powder applications of *P. fluorescens* to rice seeds followed by foliar sprays on rice shoots have resulted in the most effective reduction of blast symptoms [72]. The survival of two strains of *P. fluorescens* was examined in 3 cultivars of rice, and bacterial treatment of seeds resulted in persistence of the bacteria throughout the 110 day experiment [65]. However, the mode by which these two strains were reducing blast symptoms has not been elucidated and appears to differ from the mechanism used by EA105. While we noted elevated JA and ET signaling with minimal effect on SA, these two *Pseudomonas* isolates resulted in elevated SA levels in rice [73].

To our knowledge, this is the first report of a *Pseudomonas chlororaphis* isolate which can protect against rice blast, and this isolate shows two distinct mechanisms of action- direct antifungal activity and induction of resistance in the host. Beyond showing the ability of EA105 to inhibit vegetative growth of *M. oryzae*, we also show an ability to reduce *M. oryzae* pathogenesis by inhibiting appressoria formation. Interestingly, the activity of EA105 is largely independent of cyanide production, despite cyanide commonly being associated with biocontrol activity in Psuedomonads.

Microbes are essential for animal health and immunity, and there are compelling reasons to believe that root-associated microbes are equally important to plants as they are to animals. Plant roots encounter diverse microbial populations in soil and generate a unique ecological niche for microbes by the secretion of resources into the rhizosphere. These rhizospheric resources are limited in abundance, and some microbes have evolved antimicrobial traits to reduce competition from other microbes and to bolster the health of their plant host. However, we lack a clear understanding of the contribution conferred by individual microbial strains within a microbiome to plant growth and protection. Since biocontrol has proven to be a successful approach to crop protection, more efforts are needed to identify potential biocontrol agents from the diverse pool of rhizospheric bacteria and to understand the mechanisms by which they positively influence plant productivity.

## Conclusions

Eleven bacteria were isolated from rhizospheric rice soil and identified. Isolate EA105, *Psuedomonas chlororaphis*, showed the strongest biocontrol potential against blast pathogen *M. oryzae*. EA105 reduced mycelial growth, and almost completed halted appressoria formation in *M. oryzae*. A HCN mutant in EA105, D5, showed similar antagonistic abilities against *M. oryzae*, indicating a mechanism of action which is independent of HCN. Isolate EA105 as well as *Pantoea agglomerans* EA106 were able to reduce the number of blast lesions in rice, when roots were pre-treated with the bacteria prior to infection with *M. oryzae*. The response elicited in rice by EA105 and EA106 is mediated through JA and ET signaling. Isolate EA105 was the only isolate which was effective both as a direct antagonist to *M. oryzae* as well as an elicitor of the ISR response in rice. Isolate EA105 shows promise as a potentially valuable biocontrol agent to reduce crop losses from blast disease. The resulting increase in rice yields could have a tremendous impact on global food security.

## Materials and methods

### DNA extraction from rhizospheric soil and processing

Field grown rice plants were harvested for root associated microbial DNA for cloning and sequencing of 16S rRNA sequences. The majority of the aerial part of the rice plants was removed and a clump of soil encompassing the root ball was retained for processing. Individual roots from single plants were processed one at a time until sufficient root material was obtained for this plant. A single complete root, considered untouched during harvest, was excised from the middle of the root ball. Excess soil was removed from the root using gloved hands until only tightly bound soil remained. The root was then added to 30 ml of PBS buffer (pH 7.0). Further roots from the same plant were added until volume of roots collected approximated 12 ml. Roots in PBS buffer were vortexed, and about 16 ml of the root wash soil suspension (rice rhizosphere soil) was spun down and the pellets stored at -80 C until DNA extraction. Microbial DNA was extracted from 0.25 to 1 gram of rhizospheric soil using the MoBio UltraClean Soil DNA Isolation Kit with use of the maximum yield 'Alternative Protocol'. Amplification of 16S rDNA was performed using the primers 27 F(AGAGTTTGATCCTGGCTCAG) and 1492R (GGTTACCTTGTTACGACTT). The sequences were screened of possible chimeras using Mallard [74] and then passing sequences classified against the taxonomic reference set available from the Ribosomal Database Project (RDP) resource (http://rdp.cme.msu.edu/). Specifically, the sequences were classified using the java based RDP Naïve Bayesian rRNA Classifier Version 2.1 [75] with the taxonomic reference set RDP 10.18 [76]. The R package ggplot2 [77] was used to generate the barplots depicting taxonomic composition. The amplified product was gel purified, and cloned using the Topo TA vector. Colonies with inserts were purified, and the insert DNA sequences were obtained by Sanger sequencing.

### Isolation and identification of rhizobacteria

Natural rhizobacteria were isolated from root-associated soil and roots of M-104 rice plants, a temperate japonica cultivar widely grown in California. M-104 roots were harvested and the soil adhering to the root was removed using a sterile spatula and collected as the root-associated soil sample. The root was then rinsed, crushed and processed as the root sample, which included endophytic bacteria as well as tightly bound root bacteria. The samples were suspended in sterile water (0.1 g/ml) and serial dilutions were dispensed on LB [78], TY [79], or CP + benzoate [80] agar plates. They were incubated for 48 hours at 30°C and single colonies were selected based on morphology and re-streaked on fresh agar plates. Isolate identification was initiated by sequencing the 16S rDNA using colony PCR and the universal primers 27 F (AGAGTTTGATCCTGGCTCAG) and 1492R (GGTTACCTTGTTACGACTT). Taxonomic assignments were determined using the Ribosomal Database Project (RDP) website classifier. Further identification was done by MIDI, Inc (midi-inc.com) through a fatty acid methyl ester (FAME) analysis. A similarity (SIM) index of 1.000 means an exact species match determined by fatty acid make-up. The lower the SIM index, the more varied the fatty acid content. SIM Index cutoff of 0.600 was used to determine confident species match, unless otherwise noted.

### Plant materials and growth conditions

*Oryza sativa* 'M-104’ seeds were a gift from Dr. Thomas Tai (University of California-Davis). The seeds were dry planted in a Davis field where rice had been previously grown for several years. The field was flooded soon after emergence, and the roots were harvested for sampling at about 1 month after planting. *O. sativa* 'Maratelli’, a susceptible variety to blast fungus *M. oryzae* strain 70–15 was used for the studies. All plants were grown in a growth chamber with a daily cycle of 16 hr light (28°C, 80% RH), and 8 hr dark (26°C, 60% RH).

### In vitro antibiosis assay

Two experimental designs were created using petri dishes to determine the antagonistic activity of bacterial isolates. First is the diffusible assay, whereby sterile petri dishes were filled with autoclaved complete media (CM) agar, consisting of 10 g sucrose, 6 g yeast extract, 6 g casaminoacids, 15 g agar, and 1 ml *Aspergillus nidulans* trace elements in 1 L water. Five mm plugs of *M. oryzae* 70–15 or guy11 mycelia were placed 4 cm from 5 μl of 5 × 10^5^ bacterial cells. The plates were sealed with parafilm and put in the dark in a 25°C incubator. Photographs were taken after 5 days and the diameter of the mycelium growing out from the plug was measured using ImageJ software. Percentage (%) inhibition was calculated by the formula: % inhibition = ([C – T) × 100]/C), where C = fungal diameter (cm) in the control plate, and T = fungal diameter (cm) in the bacterial treated plates. Three biological replicates were performed and an average was taken. Second, the volatile (compartment) assay used compartmentalized petri dishes where the bacteria were grown on LB agar or LB liquid and *M. oryzae* was grown on CM agar in separate compartments. Three biological replicates were performed and an average was taken. The activated charcoal assay used the same experimental design as the volatile assay, except the remaining two compartments were each filled with 1 g of activated charcoal (Darco®, 20–40 mesh particle size, granular, Aldrich, Milwaukee, WI) wrapped in KimWipes. Two biological replicates were performed and an average was taken. For the heat killed and spent media assay, bacterial isolate EA105 was grown overnight in 10 mL of LB liquid in a 50 mL falcon tubes and optical density at 600 nm (OD_600_) was measured. The culture was either placed in a 65°C water bath for 24 hours, or spun down (centrifuged for 8 minutes at 4000 rpm) and the supernatant passed through and 0.45 μm filter (Millipore, Billerica, MA). Sterile filter discs were placed on CM agar plates 4 cm away from a 5 mm plug of *M. oryzae* 70–15. The filter discs were inoculated with 50 μl of LB liquid, 50 μl of EA105 heat-killed cells, or 50 μl of EA105 supernatant (cell-free spent media). Two biological replicates were performed and an average was taken. All fungal diameters were measured using ImageJ, and % inhibition was calculated as described above.

### Bacterial motility

To evaluate the bacterial motility, swimming and swarming assays were performed with rice isolates as per the published protocol [81]. Briefly, bacterial stabs were placed on swimming plates (5 g/L NaCl, 10 g/L tryptone, and 0.03% (w/v) agarose), and swarming plates (8 g/L nutrient broth, 5 g/L glucose, with 0.5% (wt/vol) agar and after incubation at 30°C the diameter of bacterial growth was measured.

### Measurement of cyanide

Cyanide production in bacterial culture supernatant was measured using the Lazar Model LIS-146CNCM micro cyanide ion electrode from Lazar Research Laboratories, Inc. Bacterial cultures were grown in LB for 24 hours shaking at 200 rpm at 30°C. Optical density at 600 nm (OD_600_) was recorded. The cells were centrifuged (8 minutes at 4000 rpm) and supernatant was taken for measurement. The electrode was conditioned prior to use, and rinsed with 70% ethanol then water between each sample reading. Two biological replicates were performed.

### Construction of cyanide mutant D5

The D5 mutant was constructed using the Targetron gene knockout system (Sigma-Aldrich) to disrupt a region of the hydrogen cyanide biosynthetic operon that encompassed both the *hcnB* and *hcnC* genes. Primers for the insertion sites of the group II intron were chosen by a Sigma-Aldrich computer algorithm based on an input sequence from the *hcnBC* genes. These primers (IBS, EBS1d, and EBS2) as well as the EBS universal primer were used to amplify the intron template. The resulting amplicon was purified using the QiaQuick PCR purification kit (Qiagen), double digested with HindIII and BsrGI, and then ligated into the linear pACD4K-C vector using T4 DNA ligase and 2X Rapid ligation buffer (Promega) with a 1:2 molar ratio of vector to insert DNA. Transformation was performed according to Targetron’s suggestions, with exception of the heat shock being extended to 60 seconds, the recovery period being extended to 3 hours, and the incubation temperature being at 30°C. Induction of the group II intron insertion using IPTG was performed as per the Targetron protocol. Potential transformants were selected using colony PCR and absence of cyanide production was confirmed using the LIS-146 Micro Cyanide probe (Lazar Research Laboratories).

### Solid-phase microextraction-gas chromatography mass-spectrometry (SPME-GC-MS)

Volatile metabolites produced by EA105 were extracted using an SPME fused silica fiber coated with 65 μm of polydimethylsiloxane/divinylbenzene (Sigma-Adrich). EA105 was grown on LB agar for 2 days and then the fiber was exposed for 24 hours to the headspace above EA105. The fiber was then manually injected into an Agilent 6890 GC with a 5973 N MS detector (Agilent Technologies), installed with a HP-5MS capillary column (30 m × 0.25 mm, 0.5 μm) and a flame ionization detector. Inlet temperature was 250°C. Oven conditions started at 40°C for 2 min, ramped at 10°C/min to 250°C, and held for 2 min. VOCs were identified using the mass spectral library (NIST). Standard curves of the identified compounds were created using commercially available compounds. They were diluted in methanol and 2 μl was injected into the GC. The concentration of the volatiles produced was determined by comparing peak heights of the EA105 profile to the standard curve. Four biological replicates were performed.

### Spore germination and appressoria formation

Plastic coverslips were sterilized with ethanol and used as hydrophobic surfaces for the conidiospores. *M. oryzae* 70–15 spores grown on oatmeal agar for 10 days were suspended in water and filtered through Miracloth. For S-methyl thioester treatments, a 100 mM stock of the compounds in 100% methanol was used, and compared to a control treatment with the same final amount of methanol. For cyanide treatments, potassium cyanide was dissolved in 35 mM KOH to make a 100 mM stock, which was further diluted in water. A 1:1 (v:v) solution of spores plus compound were made with a final concentration of 10^5^ spores/ml in compound concentrations ranging from 1–500 μM. For bacterial treatments, a final concentration of OD_600_ = 0.02 (~1×10^7^ cells/mL) was used. Five plastic coverslips were placed into a petri dish containing a wet filter disc in the center to maintain humidity. A 50 μL drop of treated spores was placed on each coverslip. For indirect bacterial treatment, a drop of bacterial cells was placed next to each coverslip and a 50 uL drop of untreated spores was placed on the coverslip. Petri dishes were parafilmed and placed in the dark at room temperature. Percent germination was determined at 3 hours post treatment and percent appressorium formation was determined 24 hours post treatment using the Zeiss Axioscope2 upright light microscope. Five images were taken at different locations on each coverslip for a total of 25 images per treatment. Percentage germination was calculated by counting the number of germinated spores and the total number of spores in the images. Percentage appressorium formation was determined by counting the number of germinated conidia which had produced an appressorium. Three biological replicates were examined following the protocol described above.

### Evaluation of rhizobacterial-mediated ISR

Rhizobacterial isolates were grown overnight in LB at 30°C shaking at 200 rpm. Cells were spun down by centrifugation (8 minutes at 4000 rpm) and the supernatant discarded. Cells were washed in sterile water twice, then resuspended to an OD_600_ of 0.5 (~2.5×10^8^ cells/mL). Three- week old soil-grown Maratelli rice plants were root primed with 2 mL of the rhizobacterial suspension per plant. Eight replicates were used per treatment. Mock plants were treated with 2 mL of sterile water. After 24 hours, the shoots (stems and leaves) of each plant were sprayed with 1 milliliter of *M. oryzae* strain 70–15 at a concentration of 10^5^ spores per mL. Ten-day old spores were suspended in sterile water, filtered through Miracloth, and counted using a hemocytometer. Spores were adjusted to a concentration of 1×10^5^ spores/mL water and a 1:10 (v:v) of 0.2% gelatin was added to the suspension. Plants were sprayed inside of plastic bags containing wet paper towels using an artist’s air brush, sealed to maintain humidity, and covered with plastic bins for 24 hours of darkness. As a precautionary measure, pathogen-inoculated plants were transferred to separate growth chambers and grown in identical growth conditions as the other treatment groups. Photographs of leaves were taken after 1 week and the number of lesions on the second youngest leaf was counted using the image analysis program ImageJ to facilitate accurate scoring. Four biological replicates were performed.

To test gene expression changes in rice, M-104 seeds were sterilized and germinated in petri dishes. At 7 days post germination, seedlings were transferred to clear, sterile boxes containing 50 mL of Hoagland’s liquid medium. The pH of the medium was maintained at 5.7. At 14 days post germination, the liquid medium was inoculated with bacteria which had been washed in water, to a final concentration of 10^6^ cells/mL. At 24 hours post treatment, leaf tissue was frozen in liquid nitrogen and RNA was extracted using the Bio Basic EZ-10 Spin Column Plant RNA Mini-Prep Kit. RNA was treated with Turbo DNAse (Ambion) and the High Capacity cDNA Reverse Transcription Kit (Ambion) was used to synthesize cDNA, using 500 ng of RNA. PCR was carried out using standard Taq Polymerase (New England Biolabs). Primers to test for SA responsive genes PR1 and WRKY77, JA responsive genes JAR1 and WRKY30, and ETH responsive genes EIL1 and ERF1 were designed using Primer Blast (NCBI) of Nipponbare gene sequences, and are listed in SOM Additional file 9: Table S4. PCR products were run on a 1.4% agarose gel, stained with ethidium bromide, and imaged using an Alpha Imager system. Band intensities were quantified using ImageJ. A ubiquitin control was used to normalize all samples. Each biological replicate was pooled from 9 plants, and there were 3 biological replicates per treatment.

### Statistical analysis

The statistical software JMP 10 was used to analyze data. To compare across treatments, the Tukey’s HSD test was used and results were considered to be statistically different when p < 0.05.

## Competing interests

The authors declare that they have no competing interests.

## Authors’ contributions

CS and EA isolated soil bacteria and carried out the inhibition assays. CS carried out the construction and testing of the cyanide mutant, the appressorial assays, and the gene expression assays. EA carried out the GC-MS experiments and tested the resulting compounds. CR maintained rice plants and collected rhizospheric soil samples and performed the 16S sequencing. The bioinformatic analysis of the 16S sequences was performed by CJ. CS, EA, and HB drafted the manuscript. HB conceived the study and HP, VS, and ND participated in its design and coordination. All authors read and approved the final manuscript.

## Supplementary Material

Additional file 1: Figure S1 Relative abundance (frequency) of the major bacterial genera in the rice rhizosphere microbial community recorded over a two-year period. The frequencies shown were obtained via classification of 16S rDNA sequences corresponding to a total of 654 and 630 clones, for 2008 and 2009 respectively. Click here for file

Additional file 2: Figure S2 Swimming and swarming motility of Pseudomonas isolates. Cells were grown on motility plates for 24 hours as described by Rashid & Kornberg (81). Means comparisons for all pairs were done using Tukey-Kramer HSD statistical test, where means with the same letter do not differ significantly (n=3). Treatments were compared within swarming plates, and within swimming plates. Click here for file

Additional file 3: Table S1 Comparison of fungal inhibition elicited by EA105 grown on direct or compartment plates and on agar or in liquid. Click here for file

Additional file 4: Figure S3 Growth of M. oryzae treated with heat killed cells and growth after inhibition by EA105. **A)** Effect of heat killed cells and cell-free spent media on fungal inhibition. A 50 μl drop of either heat killed EA105 cells or EA105 cell-free spent media was placed 4 cm from M. oryzae 70-15 and 70-15 diameters were measured after three days. Error bars indicate standard deviation. There was no significant difference between the control and treatments using Student’s t-test and a p-value of <0.05. **B)** Recovery of M. oryzae 70-15 growth after exposure to EA105 volatiles. Fungal plugs were replated onto fresh CM agar after previously being exposed to antifungal volatiles produced by the Pseudomonas isolate EA105. Fungal diameter was measure after three days, and normal growth was observed. There was no significant difference between the control and previously exposed 70-15. Error bars indicate standard error. Click here for file

Additional file 5: Figure S4 Activity of volatile compounds produced by bacteria in the presence of activated charcoal. Inhibitory effect through bacterial volatiles was abolished in the presence of activated charcoal. Error bars indicate standard deviation. Means with the same letter do not differ significantly as per Student’s t-test, p<0.05. Capital letters were used for plates without activated charcoal, and lower case letters were used for plates amended with activated charcoal. Click here for file

Additional file 6: Table S2 Concentration at which volatile metabolites are being produced by EA105. Click here for file

Additional file 7: Figure S5 Inhibition of M. oryzae by S methyl thioesters and 1-undecene. **A)** Standard curves used to calculate biological concentrations of volatiles produced by EA105. Commercially available compounds were diluted in methanol (S-methyl thiopropioante, S-methyl thioisovalerate), or chloroform (1-undecene) and injected into a GC-MS for analysis. **B)** Growth of M. oryzae 70-15 after 5 days on plates containing different concentrations of S-methyl thioesters in the media. Significant inhibition occurred by 1 mM for all except S-methyl thioisovalerate (Student’s t-test, p<0.05) Error bars indicate standard error. **C)** Growth of M. oryzae 70-15 after 5 days on plates containing different concentrations of 1-undecene in the media. Significant inhibition occurred by 5 mM 1-undecene (Student’s t-test, p<0.05). Error bars indicate standard error. Click here for file

Additional file 8: Table S3 Effect of treating spores with thiol-esters on germination and ability to form appresoria. Click here for file

Additional file 9: Table S4 Primer sequences used for RT-PCR gene expression in rice cv. M-104. Click here for file
