# Supplementary figures and images for: Natural rice rhizospheric microbes suppress rice blast infections
Source: BMC Plant Biol. 2014 May 13;14:130. doi: 10.1186/1471-2229-14-130 (PMC4036093; doi:10.1186/1471-2229-14-130)

Spence et al. Additional file 1: Figure S1

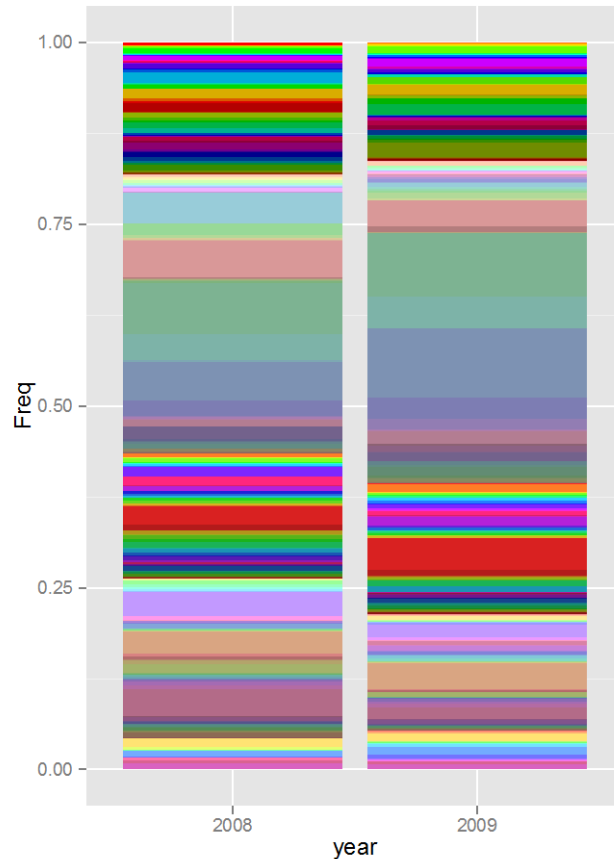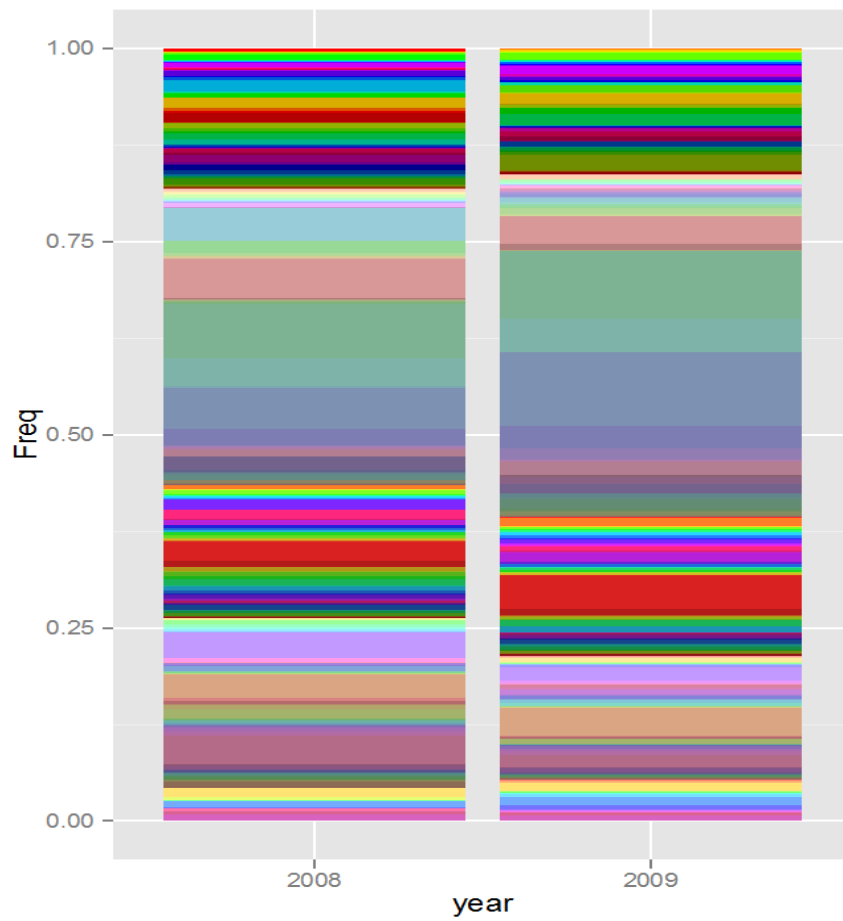

Supplement: Additional file 1: Figure S1 — Relative abundance (frequency) of the major bacterial genera in the rice rhizosphere microbial community recorded over a two-year period. The frequencies shown were obtained via classification of 16S rDNA sequences corresponding to a total of 654 and 630 clones, for 2008 and 2009 respectively. [file 1471-2229-14-130-S1.pdf]

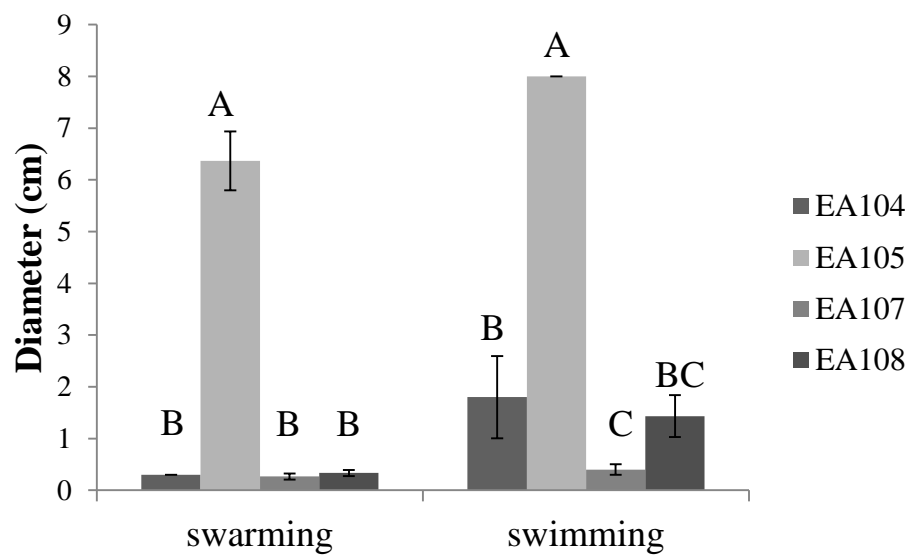

Supplement: Additional file 2: Figure S2 — Swimming and swarming motility of Pseudomonas isolates. Cells were grown on motility plates for 24 hours as described by Rashid & Kornberg (81). Means comparisons for all pairs were done using Tukey-Kramer HSD statistical test, where means with the same letter do not differ significantly (n=3). Treatments were compared within swarming plates, and within swimming plates. [file 1471-2229-14-130-S2.pdf]

**A**

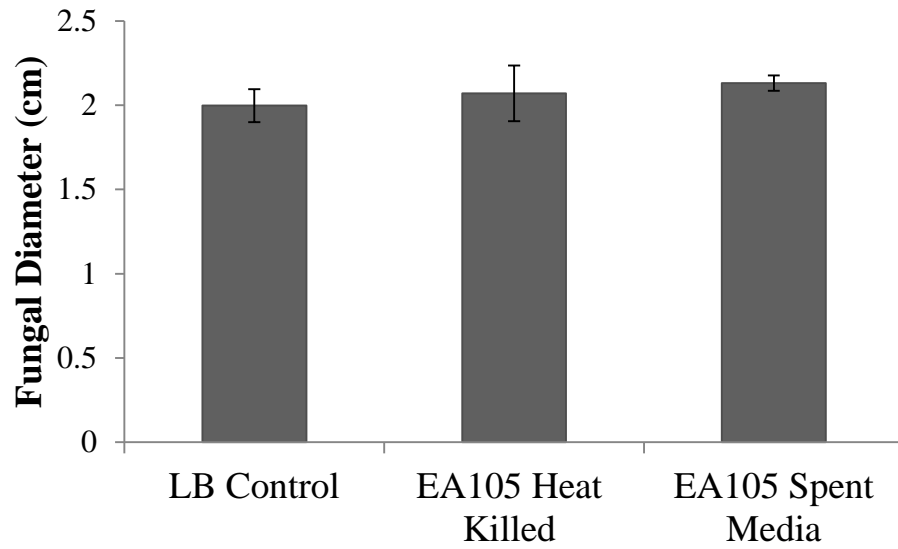

**B**

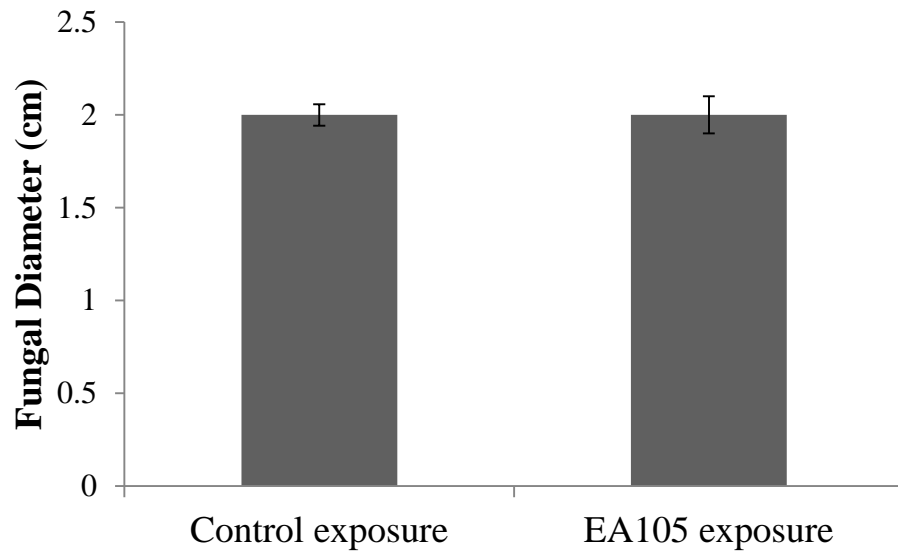

Supplement: Additional file 4: Figure S3 — Growth of M. oryzae treated with heat killed cells and growth after inhibition by EA105. A) Effect of heat killed cells and cell-free spent media on fungal inhibition. A 50 μl drop of either heat killed EA105 cells or EA105 cell-free spent media was placed 4 cm from M. oryzae 70-15 and 70-15 diameters were measured after three days. Error bars indicate standard deviation. There was no significant difference between the control and treatments using Student’s t-test and a p-value of <0.05. B) Recovery of M. oryzae 70-15 growth after exposure to EA105 volatiles. Fungal plugs were replated onto fresh CM agar after previously being exposed to antifungal volatiles produced by the Pseudomonas isolate EA105. Fungal diameter was measure after three days, and normal growth was observed. There was no significant difference between the control and previously exposed 70-15. Error bars indicate standard error. [file 1471-2229-14-130-S4.pdf]

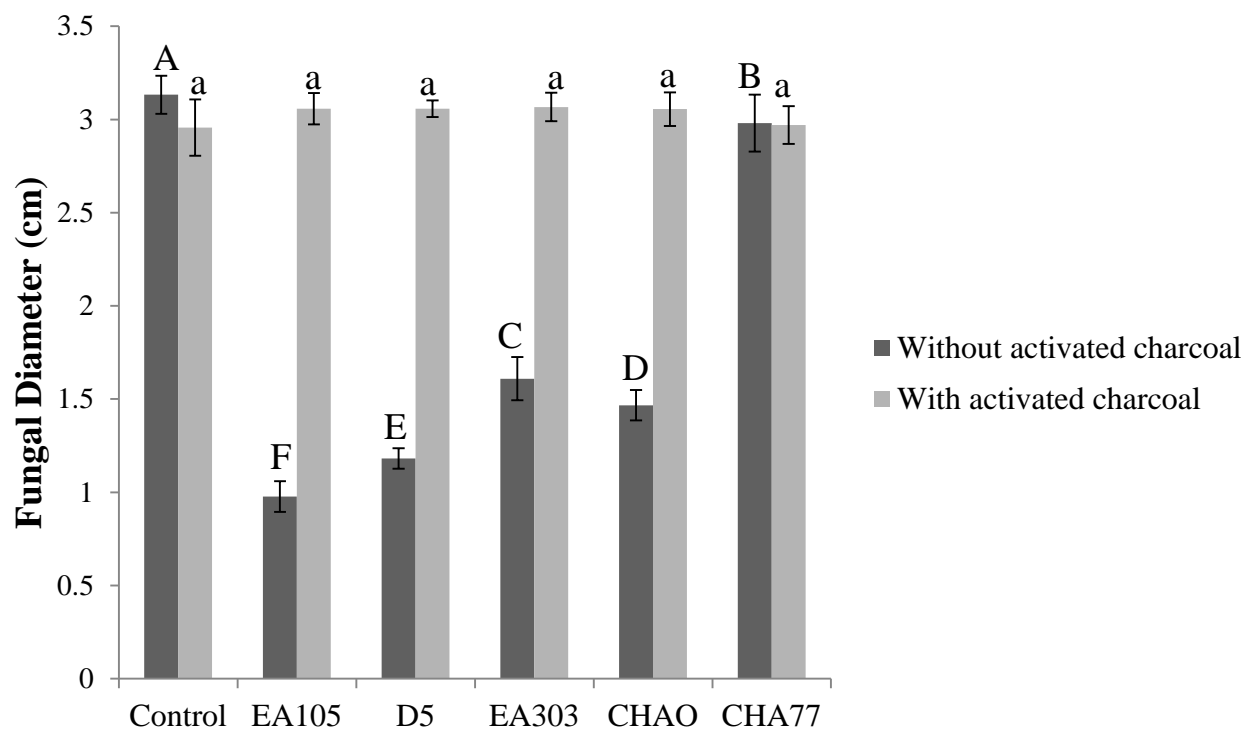

Supplement: Additional file 5: Figure S4 — Activity of volatile compounds produced by bacteria in the presence of activated charcoal. Inhibitory effect through bacterial volatiles was abolished in the presence of activated charcoal. Error bars indicate standard deviation. Means with the same letter do not differ significantly as per Student’s t-test, p<0.05. Capital letters were used for plates without activated charcoal, and lower case letters were used for plates amended with activated charcoal. [file 1471-2229-14-130-S5.pdf]

**A**

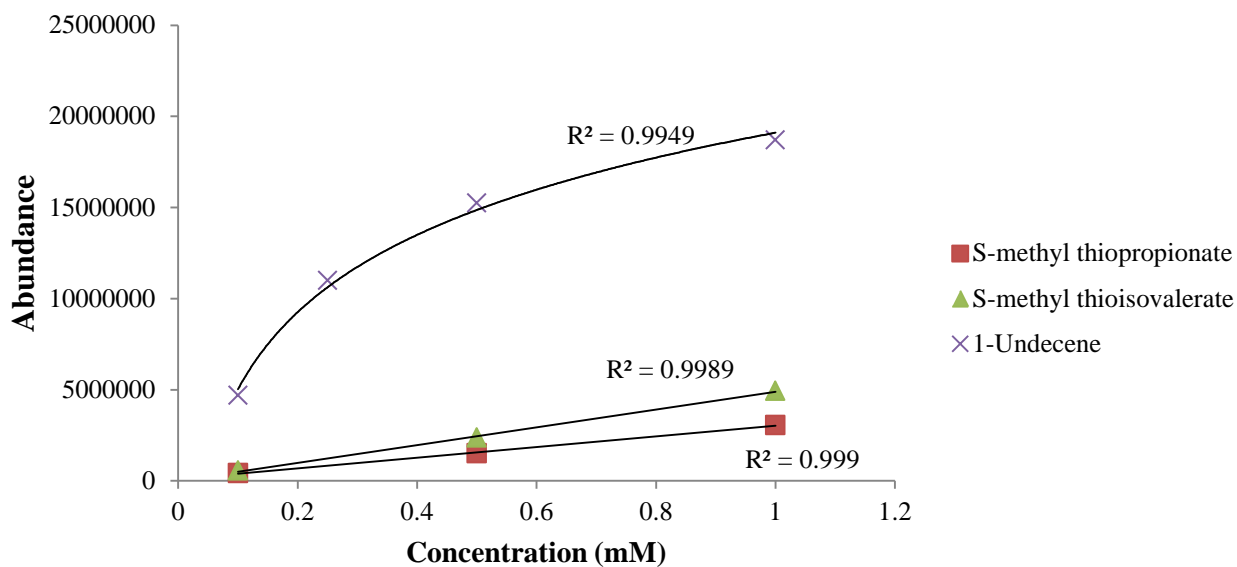

**B**

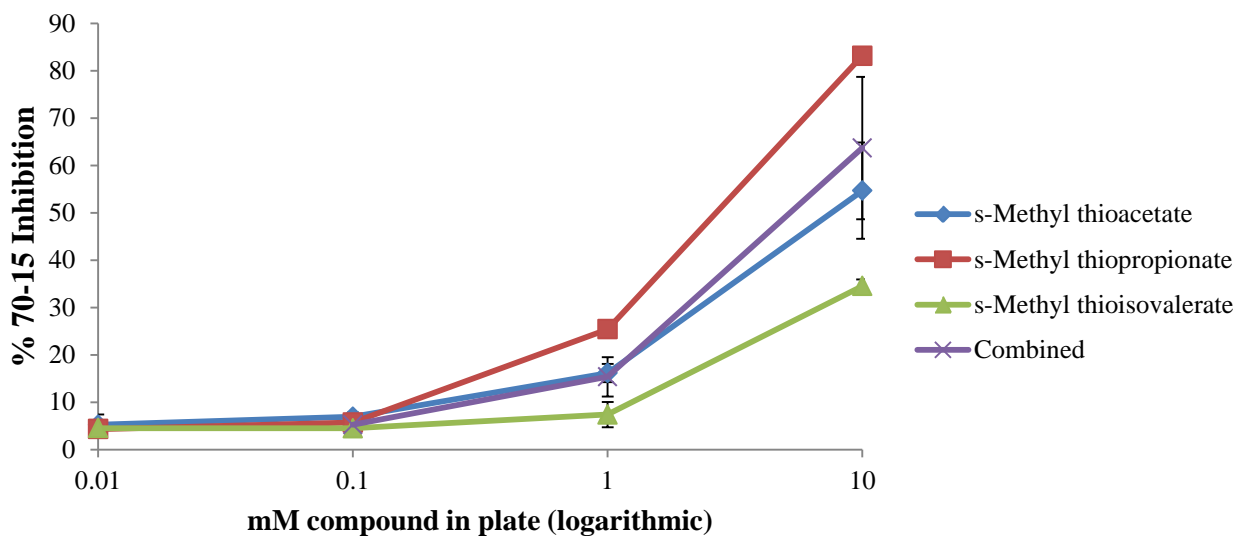

**C**

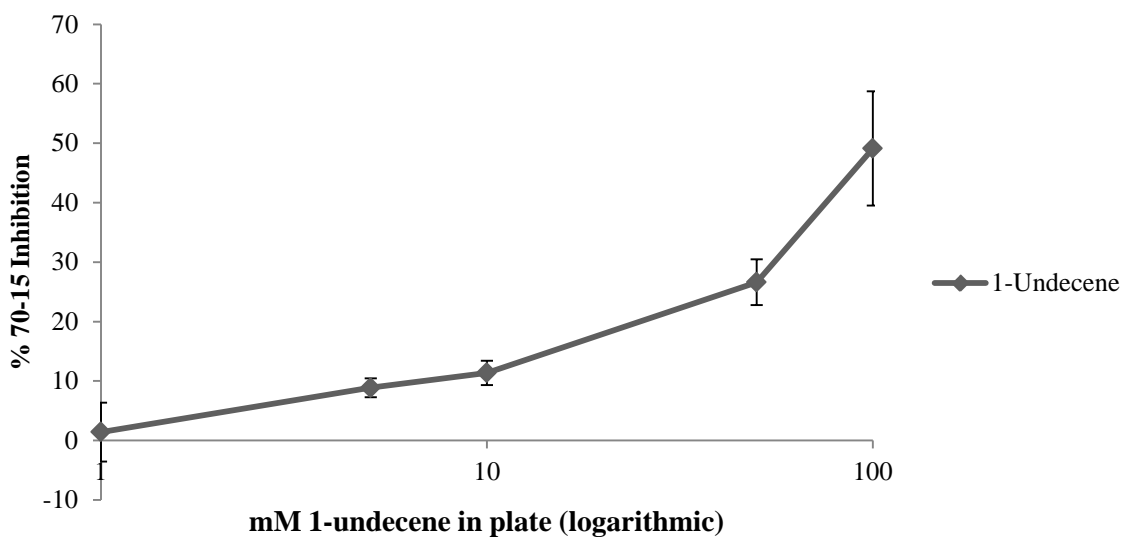

Supplement: Additional file 7: Figure S5 — Inhibition of M. oryzae by S methyl thioesters and 1-undecene. A) Standard curves used to calculate biological concentrations of volatiles produced by EA105. Commercially available compounds were diluted in methanol (S-methyl thiopropioante, S-methyl thioisovalerate), or chloroform (1-undecene) and injected into a GC-MS for analysis. B) Growth of M. oryzae 70-15 after 5 days on plates containing different concentrations of S-methyl thioesters in the media. Significant inhibition occurred by 1 mM for all except S-methyl thioisovalerate (Student’s t-test, p<0.05) Error bars indicate standard error. C) Growth of M. oryzae 70-15 after 5 days on plates containing different concentrations of 1-undecene in the media. Significant inhibition occurred by 5 mM 1-undecene (Student’s t-test, p<0.05). Error bars indicate standard error. [file 1471-2229-14-130-S7.pdf]
