# Supplementary material for: Natural rice rhizospheric microbes suppress rice blast infections
Source: BMC Plant Biol. 2014 May 13;14:130. doi: 10.1186/1471-2229-14-130 (PMC4036093; doi:10.1186/1471-2229-14-130)
Supplement: Additional file 3: Table S1 — Comparison of fungal inhibition elicited by EA105 grown on direct or compartment plates and on agar or in liquid. [file 1471-2229-14-130-S3.pdf]

Additional file6: Table S1

Additional table 1. Comparison of fungal inhibition elicited by EA105 grown on direct or compartment plates and on agar or in liquid.

	% Inhibition at 3 dpi
EA105 diffusible on CM agar	62% * (n=30)
EA105 volatile on LB agar	50% * (n=42)
EA105 volatile on CM agar	66% * (n=4)
EA105 compartment in liquid	61% * (n=5)

* Indicates significant inhibition (Student's t-test, $p<0.0001$)
