# Supplementary material for: Natural rice rhizospheric microbes suppress rice blast infections
Source: BMC Plant Biol. 2014 May 13;14:130. doi: 10.1186/1471-2229-14-130 (PMC4036093; doi:10.1186/1471-2229-14-130)
Supplement: Additional file 6: Table S2 — Concentration at which volatile metabolites are being produced by EA105. [file 1471-2229-14-130-S6.pdf]

Additional file7: Table S2

Additional table 2. Concentration at which volatile metabolites are being produced by EA105

Compound	Peak Height	Biosynthetic concentration (μM)
S-methyl thioacetate	149,550	[A]
S-methyl thiopropionate	195,230	30
S-methyl thioisovalerate	170,940	33
1-Undecene	12,169,570	270

[A] Concentration could not be obtained because the compound and solvents used eluted at the same retention time
