# Supplementary material for: Natural rice rhizospheric microbes suppress rice blast infections
Source: BMC Plant Biol. 2014 May 13;14:130. doi: 10.1186/1471-2229-14-130 (PMC4036093; doi:10.1186/1471-2229-14-130)
Supplement: Additional file 8: Table S3 — Effect of treating spores with thiol-esters on germination and ability to form appresoria. [file 1471-2229-14-130-S8.pdf]

Additional file8: Table S3

Additional table 3. Effect of treating spores with thiol-esters on germination and ability to form appressoria.

Treatment	% Germination Inhibition	% Appressorium Inhibition
10 µM		
S-methyl thioacetate	4.5	0.9
S-methyl thiopropionate	-1.6	9.7*
S-methyl thioisovalerate	0.1	10.2*
Combined	-1.2	2.1
100 µM		
S-methyl thioacetate	3.0	8.4*
S-methyl thiopropionate	-0.4	6.2*
S-methyl thioisovalerate	-0.2	11.5*
S-methyl combined	2.8	6.9*

* indicates significant inhibition (Student's t-test, $p<0.05$)
