# Supplementary material for: Natural rice rhizospheric microbes suppress rice blast infections
Source: BMC Plant Biol. 2014 May 13;14:130. doi: 10.1186/1471-2229-14-130 (PMC4036093; doi:10.1186/1471-2229-14-130)
Supplement: Additional file 9: Table S4 — Primer sequences used for RT-PCR gene expression in rice cv. M-104. [file 1471-2229-14-130-S9.pdf]

Additional file9: Table S4

Additional table 4. Primer sequences used for RT-PCR gene expression in rice cv. M-104.

Gene	Forward Primer (5` to 3`)	Reverse Primer (5` to 3`)
PR1	TCGTATGCTATGCTACGTGTTT	CACTAAGCAAATACGGCTGACA
WRKY77	CTGTGTCCAGCTACCTCTCC	TGAAGAGAGCGATCACCTC
JAR1	TCTCCCCAGCCTTAACCGTA	CTAAACGCGACGACAAACCC
WRKY30	ACTTCTTGAGTCGCCGGTTT	GCTTCTGGGATGCTCACTGT
EIL1	ATCACCAGCGCCATATCGTT	CACGGTTGTTCAGCATCAGC
ERF1	CATATCACCTTGACGCCCCA	ACCCTCACAAACTCACTCGG
